# Special Issue “Viral Infections: Physiology, Pathophysiology, Pathogenesis, Diagnosis and Treatment”

**DOI:** 10.3390/ijms27020940

**Published:** 2026-01-17

**Authors:** Barbara Bażanów, Dominika Stygar

**Affiliations:** 1 Division of Microbiology, Department of Pathology, Faculty of Veterinary Medicine, Wroclaw University of Environmental and Life Sciences, 50-375 Wroclaw, Poland; 2 Department of Physiology, Faculty of Medical Sciences in Zabrze, Medical University of Silesia, 41-808 Zabrze, Poland

Viral infections remain one of the most significant challenges to global health, affecting humans and animals alike and posing continuous threats due to their high transmissibility, genetic variability, and capacity to disrupt host homeostasis at multiple biological levels [1]. Despite remarkable advances in molecular biology, immunology, and antiviral therapy, viral diseases continue to cause substantial morbidity, mortality, and socioeconomic burden worldwide [2]. The complexity of virus–host interactions, together with the dynamic evolution of viral populations, necessitates integrated research approaches that span from fundamental molecular mechanisms to applied diagnostic and therapeutic strategies [3].

This Special Issue, “Viral Infections: Physiology, Pathophysiology, Pathogenesis, Diagnosis and Treatment”, was conceived to provide a comprehensive overview of current advances in the field of virology, highlighting how viral infections influence cellular physiology, host defense mechanisms, microbial ecosystems, and population-level dynamics. The contributions collected in this issue reflect the multifaceted nature of viral diseases and emphasize the importance of interdisciplinary research in understanding viral pathogenesis and improving disease management.

At the most fundamental level, viral infections initiate a cascade of intracellular events that determine whether the host cell successfully restricts viral replication or becomes permissive to disease progression. These early responses form the basis upon which subsequent host–virus interactions unfold, ultimately shaping disease outcomes at the organismal and population levels.

Cellular Stress Responses and Antiviral Defense Mechanisms

At the cellular level, viral infections profoundly alter host metabolic pathways and stress responses, which in turn shape the outcome of infection. One of the key processes implicated in viral pathophysiology is oxidative stress, resulting from an imbalance between reactive oxygen species production and antioxidant defenses [4]. In this Special Issue, Bażanów et al. (contribution 1) investigated the effects of different respiratory viruses on oxidative stress markers using an in vitro lung cell model. Their findings demonstrate that viral infection induces distinct oxidative stress profiles depending on both the viral agent and the cellular context. Notably, non-enzymatic oxidative stress markers were more prominently affected in lung carcinoma cells, whereas both enzymatic and non-enzymatic parameters were altered in lung fibroblasts. These observations underscore the importance of host cell type in shaping virus-induced oxidative responses and suggest that oxidative stress may contribute differently to viral pathogenesis in normal versus transformed cells.

Complementing these observations, Ou et al. (contribution 2) explored antiviral defense mechanisms mediated by heme oxygenase-1 (HO-1) during dengue virus infection. Their study revealed that sofalcone, a clinically used gastroprotective drug, suppresses dengue virus replication by activating the Nrf2/HO-1 pathway and restoring antiviral interferon responses. By linking oxidative stress regulation to innate immune signaling, this work highlights HO-1 as a critical node connecting cellular stress responses with antiviral immunity and identifies a promising candidate for drug repurposing in dengue therapy.

However, cellular antiviral responses do not operate in isolation, as infected cells are embedded within complex biological environments that can profoundly modulate host susceptibility and immune defense.

Virus–Host Interactions within Biological Ecosystems

Beyond the intracellular level, viral infections unfold within host-associated ecosystems that include diverse microbial communities and their metabolic products. Increasing evidence indicates that the microbiome plays a crucial role in modulating host susceptibility to viral infections, immune responses, and disease severity [5]. This aspect is comprehensively addressed by Hao et al. (contribution 3) in their systematic review and analysis of respiratory microbiomes in influenza compared with other respiratory infections. By synthesizing data from multiple studies, the authors demonstrate that influenza is associated with characteristic patterns of microbiome dysbiosis, including reduced microbial diversity and enrichment of specific bacterial taxa. Importantly, both shared and distinct microbiome signatures were identified across different respiratory infections, age groups, and disease severities, highlighting the bidirectional relationship between viral infection and microbial ecology.

Extending the concept of microbiome–virus interactions to antiviral intervention, Danova et al. (contribution 4) investigated the antiviral properties of Lactobacilli-derived postmetabolites against phylogenetically distant herpesviruses. Their in vitro results show that these postbiotics exert broad-spectrum antiviral effects by interfering with viral adsorption, extracellular virions, and intracellular replication stages. The study provides compelling evidence that microbial metabolites may serve as natural antiviral agents and supports the exploration of postbiotics as adjunctive or alternative strategies in the prevention and treatment of viral infections.

Beyond shaping host responses, these biological environments also influence viral replication dynamics and selective pressures, ultimately contributing to viral diversity and evolution.

Viral Diversity, Evolution, and Molecular Surveillance

The rapid evolution and genetic diversification of viruses necessitate equally dynamic and sensitive diagnostic tools capable of tracking viral variants across both clinical and population levels. High mutation rates and genomic plasticity present major challenges for disease control, diagnostics, and vaccination strategies, making molecular surveillance an essential component of modern virology. In this Special Issue, Tao et al. (contribution 5) addressed these challenges in the context of porcine reproductive and respiratory syndrome virus by developing a multiplex RT–qPCR assay capable of simultaneous virus identification and lineage typing. The assay demonstrated high sensitivity, specificity, and applicability to large numbers of clinical samples, offering a practical and efficient tool for surveillance and control of PRRSV in the swine industry. This work highlights the importance of advanced molecular diagnostics in managing viral diseases of veterinary significance. At the population level, Costa et al. (contribution 6) explored the dynamics of SARS-CoV-2 mutations using wastewater-based epidemiology. By combining nested PCR with next-generation sequencing of selected spike gene regions, the authors successfully detected and quantified variant-associated mutations in wastewater samples. Notably, some mutations corresponding to variants of concern were identified prior to their widespread detection in clinical samples, underscoring the value of environmental surveillance as an early warning system for emerging viral variants.

Accurate detection and surveillance of viral variants not only inform epidemiological control but also provide critical guidance for the development and application of effective antiviral therapies.

Antiviral Strategies and Therapeutic Targeting

The continuous emergence of drug-resistant viral strains and the limited availability of effective antiviral therapies underscore the urgent need for new antiviral agents with novel mechanisms of action [6]. Several contributions to this Special Issue address this challenge by exploring diverse antiviral strategies that target both viral components and host pathways. Cho and Ma (contribution 7) demonstrated the antiviral activity of conessine, a steroidal alkaloid of plant origin, against influenza A virus. Their results indicate that conessine interferes with early stages of viral infection, including viral attachment and entry, and exhibits a direct virus-eradicating effect. By targeting host–virus interactions rather than viral enzymes alone, conessine represents a promising candidate for the development of alternative anti-influenza therapies. Together with the mechanistic insights provided by Ou et al. on HO-1-mediated antiviral responses, these studies highlight the diversity of therapeutic strategies currently being explored, ranging from natural compounds to host-directed antiviral interventions. As the search for novel antiviral strategies intensifies, the reliability of experimental models and analytical tools becomes increasingly critical to ensure that therapeutic advances are built on robust and reproducible data.

Methodological Challenges in Virology Research

Robust methodology and critical data interpretation are fundamental to advancing virology research. In this Special Issue, Ripa et al. (contribution 8) address an important methodological concern related to the use of LC3 immunofluorescence as a marker of autophagy in herpes simplex virus type 1-infected cells. Their work demonstrates that polyclonal LC3B antibodies can produce non-specific nuclear staining, potentially leading to misinterpretation of autophagy activation during viral infection. By systematically validating their observations using complementary approaches, the authors underscore the necessity of methodological rigor and cross-validation in experimental virology. This contribution serves as an important reminder that careful evaluation of experimental tools is essential to ensure the reliability and reproducibility of conclusions drawn from virological studies. Together, these considerations highlight that progress in virology depends not only on innovative concepts and technologies, but also on careful validation and critical interpretation of experimental evidence.

Conclusions and Future Perspectives

The articles collected in this Special Issue provide a multifaceted view of viral infections, spanning cellular stress responses, host–microbiome interactions, viral evolution, diagnostic innovation, therapeutic development, and methodological considerations. These interconnected processes are summarized schematically in Figure 1, which illustrates viral infections as dynamic, multi-layered phenomena extending from intracellular events to population-level surveillance and intervention strategies. Future research in virology will benefit from increasingly integrative approaches that combine basic and translational science, leverage advanced molecular technologies, and emphasize methodological robustness. By bridging physiology, pathophysiology, diagnostics, and treatment, the studies presented in this Special Issue contribute valuable insights that advance our understanding of viral infections and support the development of more effective strategies for their control and management.

## Figures and Tables

**Figure 1:**
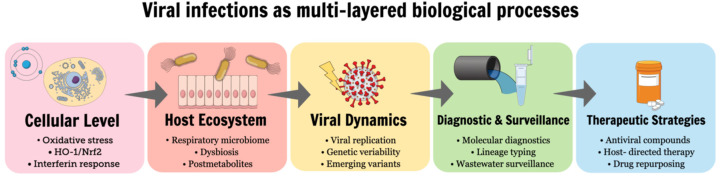
Conceptual overview of the multi-layered processes involved in viral infections addressed in this Special Issue. Viral infections are depicted as dynamic processes affecting host homeostasis at multiple biological levels, ranging from cellular stress responses and innate immunity, through host–microbiome interactions and viral genetic variability, to molecular diagnostics, population-level surveillance, and antiviral therapeutic strategies.
